# A transcription factor network responsive to high CO_2_/hypoxia is involved in deastringency in persimmon fruit

**DOI:** 10.1093/jxb/ery028

**Published:** 2018-01-30

**Authors:** Qing-gang Zhu, Zi-yuan Gong, Miao-miao Wang, Xian Li, Donald Grierson, Xue-ren Yin, Kun-song Chen

**Affiliations:** 1 Zhejiang Provincial Key Laboratory of Horticultural Plant Integrative Biology, Zhejiang University, Zijingang Campus, Hangzhou, PR China; 2 The State Agriculture Ministry Laboratory of Horticultural Plant Growth, Development and Quality Improvement, Zhejiang University, Zijingang Campus, Hangzhou, PR China; 3 Plant & Crop Sciences Division, School of Biosciences, University of Nottingham, Sutton Bonington Campus, Loughborough, UK

**Keywords:** Astringency removal, *ERF*, high CO_2_, hypoxia, *MYB*, persimmon fruit, transcriptional regulation

## Abstract

Plant responses to anaerobic environments are regulated by ethylene-response factors (ERFs) in both vegetative and productive organs, but the roles of other transcription factors (TFs) in hypoxia responses are poorly understood. In this study, eight TFs (*DkbHLH1*, *DkMYB9*/*10*/*11*, *DkRH2-1*, *DkGT3-1*, *DkAN1-1*, *DkHSF1*) were shown to be strongly up-regulated by an artificial high-CO_2_ atmosphere (1% O_2_ and 95% CO_2_). Dual-luciferase assays indicated that some TFs were activators of previously characterized *DkERF*s, including DkMYB10 for the *DkERF9* promoter, DkERF18/19 and DkMYB6 for the *DkERF19* promoter, and DkERF21/22 for the *DkERF10* promoter. Yeast one-hybrid and *cis*-element mutagenesis confirmed these physical interactions with one exception. The potential roles of these TFs in persimmon fruit deastringency were analysed by investigating their transient over-expression (TOX) in persimmon fruit discs, which indicated that *DkMYB6*_TOX_, *DkMYB10*_TOX_, *DkERF18*_TOX_, and *DkERF19*_TOX_ were all effective in causing insolubilization of tannins, concomitantly with the up-regulation of the corresponding genes. These results indicated that multiple TFs of different classes are responsive to high-CO_2_/hypoxia in fruit tissues, and that a TF–TF regulatory cascade is involved in the hypoxia responses involving the Group VII *DkERF10*, and *DkERF*s and *DkMYB*s.

## Teaser

High-CO_2_ treatment (95% CO_2_, 1% O_2_) triggers deastringency in persimmon fruit via a cascade of interactions between several different classes of transcription factors leading to activation of genes required for anaerobic metabolism.

## Introduction

Anoxia is a common abiotic stress for plants, usually caused by flooding (Yang *et al.*, 2011). The response to anoxia involves a range of metabolic and morphological responses over different timescales, including a rapid induction of anaerobic metabolism (Kennedy *et al.*, 1992; Voesenek and Bailey-Serres, 2015). Controlled-atmosphere storage in artificially reduced oxygen, usually supplemented with CO_2_, has been used for a long time to actively extend post-harvest storage and alleviate physiological disorders for various fruits (Ali *et al.*, 2016; Bekele *et al.*, 2016; Matityahu *et al.*, 2016) and can induce anaerobic responses. A specific benefit for fruit quality conferred by a low-O_2_ environment has been reported for astringent-type persimmon (*Diospyros kaki*) incubated in an atmosphere of 1% O_2_ and 95% CO_2_ (Pesis and Ben-Arie, 1984; Taira *et al.*, 1992, 2001; Min *et al.*, 2012). The low-oxygen environment leads to acetaldehyde accumulation, which removes astringency in persimmon fruit by precipitation of soluble tannins (Taira *et al.*, 2001; Salvador *et al.*, 2007). Controlled atmospheres containing ethylene also promote deastringency, suggesting that ethylene signaling is involved (Ikegami *et al.*, 2007; Min *et al.*, 2012, 2014; Yin *et al.*, 2012). Despite the fact that the commercial application of reduced oxygen for transportation and storage of fruit and some other plant products underpins a major industry and is important for food security, the underlying molecular mechanisms of fruit response to hypoxia are poorly understood.

In recent years, our knowledge of transcriptional regulatory mechanisms controlling hypoxia responses has been advanced significantly by the characterization of subfamily VII of the ethylene-response factors (ERF VII) (Sasidharan and Mustroph, 2011; Xie *et al.*, 2016). In Arabidopsis, five *ERF* genes, namely hypoxia-responsive *ERF1* (*HRE1*), *HRE2*, *RAP2.2*, *RAP2.3*, and *RAP2.12*, have been reported as the main plant oxygen-sensing regulators, and have been shown to control fermentation-related *ADH* and *PDC* genes (Hinz *et al.*, 2010; Licausi *et al.*, 2010; Yang *et al.*, 2011; Bui *et al.*, 2015; Papdi *et al.*, 2015). This sensing system operates via the N-end rule pathway, which controls plant *ERF* hypoxia responses, via post-translational regulation (Gibbs *et al.*, 2011; Licausi *et al.*, 2011*a*). Involvement of *ERF*s in the regulation of hypoxia responses has also been reported in other plants, such as rice submergence tolerance-related *Submergence 1* (*Sub1*; Xu *et al.*, 2006), *ERF VII* in *Rumex* and *Rorippa* (van Veen *et al.*, 2014), and *ERF VII* in apple fruit (Cukrov *et al.*, 2016). Potential roles for *ERF*s in persimmon fruit responses to hypoxia have also been investigated. Eighteen *DkERF* genes were shown to be responsive to treatment with 95% CO_2_ (1% O_2_), but only *DkERF9*, *10*, *19*, and *22* were capable of trans-activation of the promoters of *DkADH* and *DkPDC* genes (Min *et al.*, 2012, 2014). Moreover, of these four *DkERF* genes, only *DkERF10*, which has similarity to Arabidopsis *HRE2*, belongs to subfamily VII, indicating either that the hypoxia response is more complicated than revealed by investigations in Arabidopsis or that the ERF-VIIs may be regulated mainly at the post-translational level.

*ERF*s are one of the most comprehensively investigated transcription factor (TF) families with regards to involvement in plant hypoxia responses, although a few other hypoxia-related TFs have been reported, such as Arabidopsis *AtMYB2* (Hoeren *et al.*, 1998) and *Heat shock factor A2* (*HsfA2*; Banti *et al.*, 2010), wheat *TaMYB1* (Lee *et al.*, 2007), persimmon *DkMYB6* (Fang *et al.*, 2016) and *DkTGA1* (Zhu *et al.*, 2016). Omics-based analyses, however, have indicated many more TFs are responsive to hypoxia; for instance, at least 22 *ERF*s are regulated by anoxia in coleoptiles of rice (Lasanthi-Kudahettige *et al.*, 2007), and additional differentially expressed TFs have been characterized from Arabidopsis roots, leaves, and seedlings (Branco-Price *et al.*, 2005; Liu *et al.*, 2005; Mustroph *et al.*, 2009; Lee *et al.*, 2011; Licausi *et al.*, 2011*b*). These data indicated the involvement of a variety of TFs in hypoxia responses in plants, although whether and how they interact is unclear.

In the present research, using astringency loss as a reporter of the anaerobic response, we utilized RNA-seq data previously used for *DkERF* isolation (Min *et al.*, 2012, 2014) and identified unigenes for TFs that were up-regulated by an artificial high-CO_2_ atmosphere (AHCA; 1% O_2_ and 95% CO_2_). Another treatment, AHNA (artificial high-N_2_ atmosphere; 99% N_2_ and 1% O_2_) was introduced to distinguish between responses to high CO_2_ and hypoxia. Both high-CO_2_-responsive and hypoxia-responsive TFs were selected for further analyses. Regulatory interactions of these TFs during hypoxia-triggered deastringency in persimmon fruit were investigated by dual-luciferase assays, yeast one-hybrid interactions, and promoter motif mutations. In the absence of a transformation system for persimmon, the functions of some potential regulators were analysed by transient over-expression in persimmon fruit discs.

## Materials and methods

### Plant material and treatments

Three astringent-type persimmon (*Diospyros kaki*) fruit were selected for this study, namely two Chinese cultivars, ‘Mopanshi’ and ‘Jingmianshi’, and one Japanese cultivar, ‘Tonewase’, all of which were collected from an orchard at Qingdao (Shandong, China) in 2014. Fruit without disease or signs of mechanical wounding were selected and divided into two batches: (1) the first batch was treated with AHCA (artificial high-CO_2_ atmosphere, 1% O_2_ and 95% CO_2_) in sealed in air-tight containers for 1 d to remove astringency, and (2) the second batch was sealed in similar containers containing air for 1 d, as a control.

In order to distinguish between the effects of high CO_2_ and low oxygen, AHNA (artificial high-N_2_ atmosphere, 99% N_2_ and 1% O_2_) treatments were performed using the cultivar ‘Gong cheng-shui shi’, which was obtained from a commercial orchard at Gongcheng (Guilin, China) in 2017. The fruit were divided into three batches: (1) the first batch was treated with AHCA in sealed air-tight containers for 1 d, (2) the second batch was treated with AHNA in similar containers, and (3) the third batch was sealed in containers containing air, as a control.

The treated fruit were transferred to storage in air at 20 °C. Fruit flesh from three replicate samples each of which consisted of four fruit were sampled for each treatment at all sampling points. The samples were frozen in liquid nitrogen and stored at –80 °C until further use.

### Soluble condensed tannins

Soluble condensed tannins are the main source of astringency for persimmon fruit. Here, two different methods were selected to determine the content of soluble condensed tannins. The printing method was used for fruit flesh, according to Min *et al.*, (2015). The whole fruit (1 d after picking, immediately after treatments) was cut into two parts and the cut surface was printed onto processed filter paper that had been soaked with 5% FeCl_2_ and then oven-dried at 60 °C. The content of soluble tannins was indicated by the intensity of the black color on the filter paper.

A more accurate measure of the content of soluble tannins from frozen samples was obtained with Folin–Ciocalteu reagent, with three biological replicates, according to the method described by Yin *et al.*, (2012). The results were calculated using a standard curve of tannin acid equivalents.

### Acetaldehyde and ethanol

Acetaldehyde and ethanol production were measured with a gas chromatograph (Agilent 6890N, USA), fitted with a FID column (HP-INNOWAX, 0.25 mm, 30 m, 0.25 μm, Agilent J&W, CA, USA), using the same parameters described previously by Min *et al.* (2012). In brief, 2 g frozen fruit flesh was ground in liquid nitrogen and added to 5 ml saturated NaCl solution. Then 3 ml of the mixture was transferred to 10-ml air-tight vials with crimp-top caps. The vials were placed in a water-bath at 60 °C for 1 h, after which 0.2 ml of head-space gas was removed for analysis. The injector, detector, and oven temperatures were set at 150, 160 and 100 °C, respectively. Sec-butyl alcohol (Sigma) was used as an internal control. The results were calculated using standard curves for acetaldehyde and ethanol. All measurements were conducted with three biological replicates.

### RNA extraction and cDNA synthesis

Total RNA was extracted from frozen persimmon fruit flesh samples (2.0 g for each), using the method described by Chang *et al.* (1993). The total RNA was treated with a TURBO DNA-free kit (Ambion) to remove the genomic DNA. First-strand cDNA synthesis was initiated from 1.0 μg DNA-free RNA, using an iScript^TM^ cDNA Synthesis Kit (Bio-Rad). For each sampling point, three biological replicates were used for RNA extraction and the subsequent cDNA synthesis.

### Gene isolation and sequence analysis

Using the same RNA-seq results described by Min *et al.* (2014), predicted TF-related hypoxia-responsive unigenes were isolated. The UTR regions of the transcripts were obtained using a SMART RACE cDNA amplification Kit (Clontech) and the primers are described in Supplementary Tables S1 and S2 at *JXB* online. The sequences of full-length TFs were confirmed and amplified with primers spanning the start and stop codons (Supplementary Table S3) and translated with the ExPASy software (http://web.expasy.org/translate). The newly isolated TFs were named after a BLAST analysis in Genbank and comparison with the reported TFs in persimmon.

### Real-time PCR analysis

For real-time PCR, gene-specific oligonucleotide primers were designed (see Supplementary Table S4). The quality and specificity of each pair of primers were checked by melting curves and product resequencing. The housekeeping gene *DkACT* (Min *et al.*, 2012) was chosen as the internal control and the 2^–△△Ct^ method was used to calculate the relative expression levels of genes (Livak and Schmittgen, 2001). The expression at the time-point of fruit harvest (0 d) was set as 1 for each gene.

PCR reactions were performed on a CFX96^TM^ Real-Time System (Bio-Rad). PCR reaction mixtures (20 μl) comprised 10 μl of SsoFast^TM^ EvaGreen Supermix (Bio-Rad), 1 μl of each primer (10 μM), 2 μl diluted cDNA, and 6 μl DEPC-treated water. The PCR program was initiated with a preliminary step of 30 s at 95 °C, followed by 45 cycles of 95 °C for 5 s, 60 °C for 5 s, and completed with a melting-curve analysis program. For real-time PCR, three biological replicates were conducted for each gene at each sampling point of each treatment.

### Dual-luciferase assay

The trans-activation by the TFs of genes related to deastringency was investigated by dual luciferase assays (Hellens *et al.*, 2005). All constructs were electroporated into *Agrobacterium tumefaciens* GV3101. Full-length TFs were cloned into pGreen II 002962-SK vector (SK), using the primers described in Supplementary Table S5. The promoters of alcoholic fermentation-related genes (*DkADH1* and *DkPDC2*) and deastringency-related *ERF*s (*DkERF9*, *DkERF10*, and *DkERF19*) were originally constructed by Min *et al.* (2012) and Fang *et al.* (2016), and were inserted into the pGreen II 0800-LUC vector.

The dual-luciferase assays were performed with *Nicotiana benthamiana* leaves, using the protocol described by Min *et al.* (2012, 2014). *Agrobacterium* carrying constructs were suspended in infiltration buffer (10 mM MES, 10 mM MgCl_2_. 150 mM acetosyringone, pH5.6) to an OD_600_ of approximately 0.75. TFs and promoters were combined at a ratio of 10:1 (v/v) and infiltrated into tobacco leaves by needleless syringes. Three days after infiltration, leaf discs were punched and assayed with dual-luciferase assay reagents (Promega). Dual-luciferase assays were performed with at least three independent experiments, with five biological replicates in each experiment.

### Yeast one-hybrid assay

Yeast one-hybrid assays (Y1Hs) were performed in order to verify the gene–gene interactions, using the Matchmaker^TM^ Gold Yeast One-Hybrid Library Screening System (Clontech, USA). The full-length *DkMYB10* was subcloned into the pGADT7 AD vector and the promoter of *DkERF9* was constructed into the pAbAi vector according to the ClonExpress II One-Step Cloning Kit (Vazyme, Nanjing) (primers are listed in Supplementary Table S6). Auto-activation and the interaction analyses were conducted according to the manufacturer’s protocol.

### Site-directed mutagenesis of gene promoters

Due to auto-activation of the promoters of *DkERF10* and *DkERF19* in yeast (see Supplementary Fig. S7), site-directed mutagenesis was performed for the *DkERF9*, *DkERF10*, and *DkERF19* promoters to eliminate the predicted binding sites for ERF and MYB TF (see Results). Motif mutations were carried out using the Fast Mutagenesis System (Transgene, Beijing) (primers are listed in Supplementary Table S7). Trans-activation effects of TFs on mutated promoters were further analysed by dual-luciferase assays.

### Transient overexpression in persimmon fruit discs

In order to further verify the potential roles of TFs in persimmon fruit deastringency, transient overexpression (TOX) was performed with persimmon fruit discs. Discs of 1 cm diameter and 0.5 cm thickness were divided into five batches. The discs were incubated for 1 h with *Agrobacterium* carrying constructs in the same buffer used for the dual-luciferase assay. The discs were then transferred to filter papers (wetted by Murashige and Skoog medium) in tissue-culture dishes, and placed in an incubator at 25 °C for 3 d. All of the experiments (all genes and the empty vector) were performed with three biological replicates. At each sampling point (each day), the discs were dried on filter papers, frozen in liquid nitrogen and stored at –80 °C for further use.

### Statistical analysis

The statistical significance of differences was determined using Student’s *t*-test by DPS2.05 (Zhejiang University, Hangzhou, China).

## Results

### Isolation and characterization of deastringency/hypoxia-responsive transcription factors from ‘Mopanshi’ persimmon fruit

From RNA-seq data from the ‘Mopanshi’ cultivar (Min *et al.*, 2014), 13 full-length TFs were amplified by RACE and designated according to blast analysis as: *DkbHLH1* and *2* (basic/helix-loop-helix, KY849612-3), *DkMYB9*, *10*, *11*, *12*, and *13* (KY849603-7), *DkRH2-1* and *2* (ring-H2 finger protein, KY849614-5), *DkGT3-1* (trihelix transcription factor GT-3, KY849616), *DkAN1-1* (zinc finger AN1 domain-containing protein, KY849617), *DkHSF1* (heat shock factor, KY849619), and *DkIAA1* (auxin-responsive protein, KY849618). These, together with previously reported TFs from persimmon (*DkERF*s, *DkNAC*s, *DkMYB6*, *DkTGA1*) (Min *et al.*, 2012, 2014, 2015; Fang *et al.*, 2016; Zhu *et al.*, 2016), were used to study transcriptional interactions in anaerobic persimmon fruit. A summary of AHCA-responsive transcription factors from persimmon fruit is given in Supplementary Table S8.

**Fig. 1.**
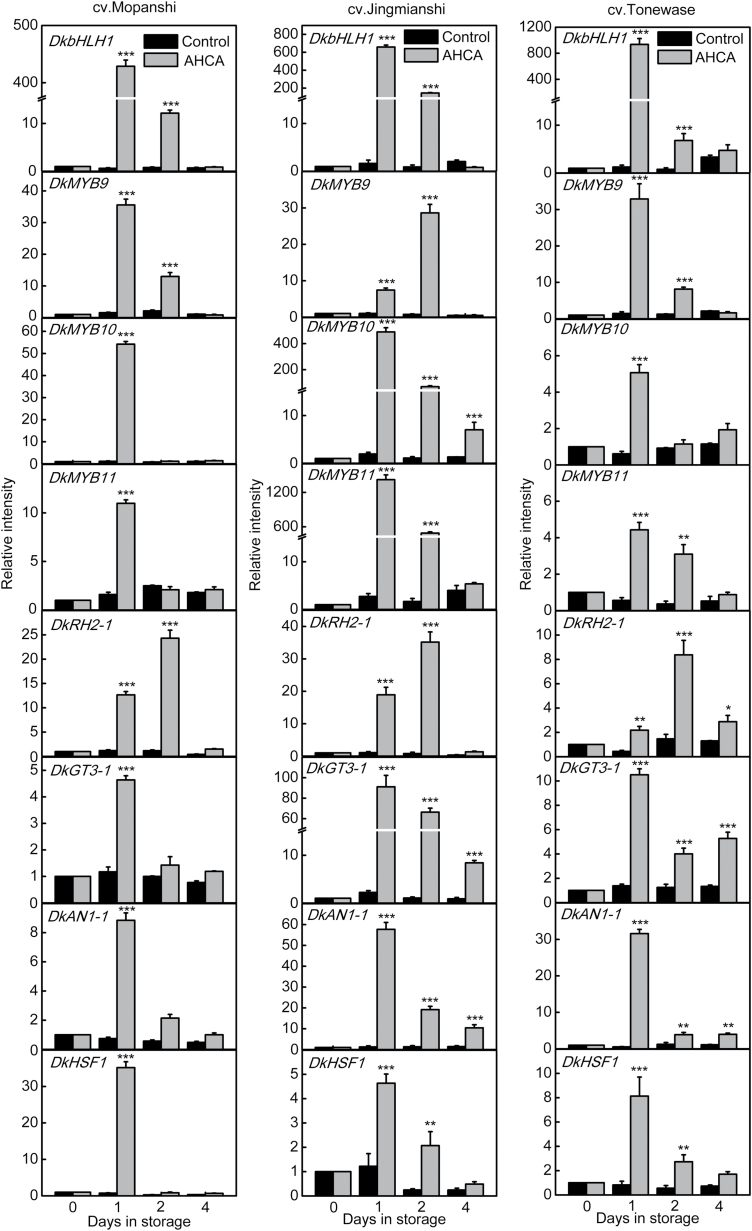
Expression of TFs in response to treatment with artificial high-CO_2_ atmosphere (AHCA, 95% CO_2_ and 1% O_2_, 1 d) in persimmon fruit cultivars ‘Mopanshi’, ‘Jingmianshi’, and ‘Tonewase’ at 20 °C. For relative mRNA abundance of the genes, the values at day 0 were set as 1. Values are means (+SE) from three biological replicates (*P<0.05, **P<0.01, ***P<0.001).

AHCA accelerated deastringency in persimmon fruit and triggered anaerobic fermentation, as indicated by bursts of acetaldehyde and ethanol production (see Supplementary Fig. S1). Expression of the 13 full-length TFs, which were predicted by RNA-seq, were analysed by real-time PCR. Eight genes were AHCA-responsive in ‘Mopanshi’, namely *DkbHLH1*, *DkMYB9*,*10*,*11*, *DkRH2-1*, *DkGT3-1*, *DkAN1-1*, and *DkHSF1* (Fig. 1). Of these, *DkbHLH1* showed the most striking response, increasing by about 429-fold after 1 d AHCA treatment, followed by *DkMYB10*, which increased by approximately 55-fold after 1 d. In ‘Jingmianshi’, *DkMYB11* was the most responsive to high-CO_2_ treatment, increasing by about 1422-fold after 1 d, followed by *DkbHLH1* and *DkMYB10*, with 658-fold and 489-fold increases, respectively. In ‘Tonewase’ only *DkbHLH1* expression was very strongly responsive to AHCA treatment, increasing by about 935-fold after 1d. In contrast, the expression of the other five of the 13 TFs showed limited responses to AHCA treatment at 1 d (Supplementary Fig. S2), at which time the content of soluble tannins had almost reached its lowest level Supplementary Fig. S1). Thus, the subsequent responses (from 2 d onwards) of these genes were probably not related to deastringency.

In addition, a comparison was made between AHCA and AHNA using the cultivar ‘Gong cheng-shui shi’ (see Supplementary Fig. S3). Among the eight AHCA-responsive TFs, five (*DkbHLH1*, *DkMYB9*, *DkMYB11*, *DkRH2-1*, and *DkHSF1*) were responsive to both AHCA and AHNA, and thus can be termed as hypoxia-responsive; the expression of the other three TFs, *DkGT3-1*, *DkAN1-1*, and *DkMYB10*, remained constant in response to AHNA, and thus these genes were responsive to high CO_2_ (Supplementary Fig. S4).

### Effect of high-CO_2_/hypoxia-responsive TFs on *DkADH* and *DkPDC* promoters

**Fig. 2.**
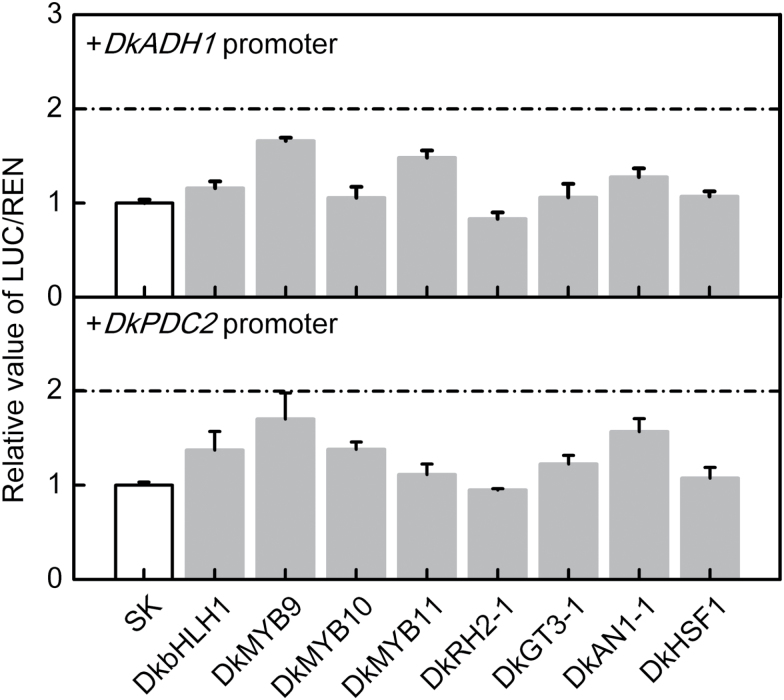
Regulatory effects of artificial high-CO_2_ atmosphere (AHCA)-responsive TFs on the promoters of *DkADH1* and *DkPDC2* determined using the dual-luciferase assay. The ratio of LUC/REN of the empty vector (SK) plus promoter was used as the calibrator (set as 1). Values are means (+SE) from five biological replicates.

The persimmon genes *DkADH1* and *DkPDC2* were previously shown to be involved in fruit deastringency and to be induced by AHCA treatment (Min *et al.*, 2012; Mo *et al.*, 2016), and *DkERF9* and *DkERF10* were shown to have direct interactions with the *DkADH1* and *DkPDC2* promoters, respectively. In order to investigate the possible roles of other hypoxia-responsive TFs, the promoters of *DkADH1* and *DkPDC2* were used for dual-luciferase trans-activation assays. The eight AHCA-responsive TFs had either limited or no effects on the *DkADH1* and *DkPDC2* promoters (less than 2-fold increase) (Fig. 2), suggesting that there is no direct regulation by any of these TFs on the promoters of *DkADH1* and *DkPDC2*.

### Relationship between high-CO_2_/hypoxia-responsive TFs

**Fig. 3.**
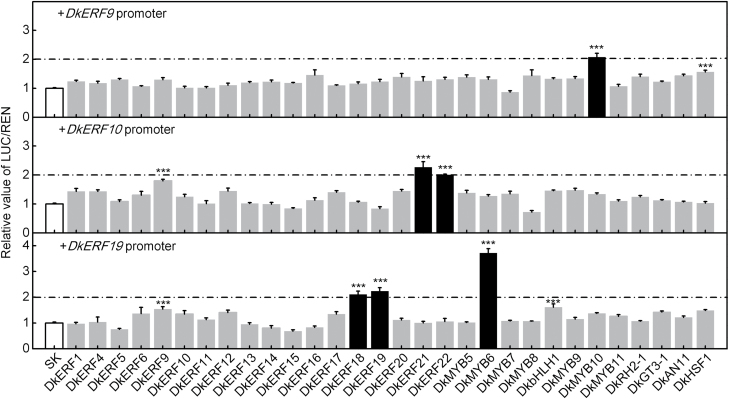
Regulatory effects of artificial high-CO_2_ atmosphere (AHCA)-responsive TFs on the promoters of deastringency-related *DkERF9*, *DkERF10*, and *DkERF19* determined using the dual-luciferase assay. AHCA-responsive *DkERF* and *DkMYB5-8* were isolated by Min *et al.* (2012) and Fang *et al.* (2016). The ratio of LUC/REN of the empty vector (SK) plus promoter was used as the calibrator (set as 1). Black columns highlight inductions of at least 2-fold. Values are means (+SE) from five biological replicates (***P<0.001).

Four *DkERF* genes, *DkERF9*,*10*,*19*, and *22*, were characterized previously as regulators of post-harvest deastringency in persimmon (Min *et al.*, 2012, 2014). A further investigation was conducted to test the possible interaction between hypoxia-responsive TFs, including 18 additional *DkERF*s and four *DkMYB*s reported previously (Min *et al.*, 2012, 2014; Fang *et al.*, 2016), and promoters of *DkERF9*,*10*, and *19*. Dual-luciferase assays indicated various trans-activation reactions, for example between DkMYB10 and the *DkERF9* promoter (approximately 2.1-fold response), DkERF21 and 22 and the *DkERF10* promoter (approximately 2.3- and 2.0-fold, respectively), and DkERF18 and 19 and DkMYB6 and the *DkERF19* promoter (approximately 2.1-, 2.2-, and 3.7-fold, respectively) (Fig. 3). The synergistic effects of DkERF21 and DkERF22 on the promoter of *DkERF10*, and DkERF18, DkERF19, and DkMYB6 on the promoter of *DkERF19* were also investigated, but there were no additive effects of these TFs on their corresponding target promoters (see Supplementary Figs S5 and S6).

**Fig. 4.**
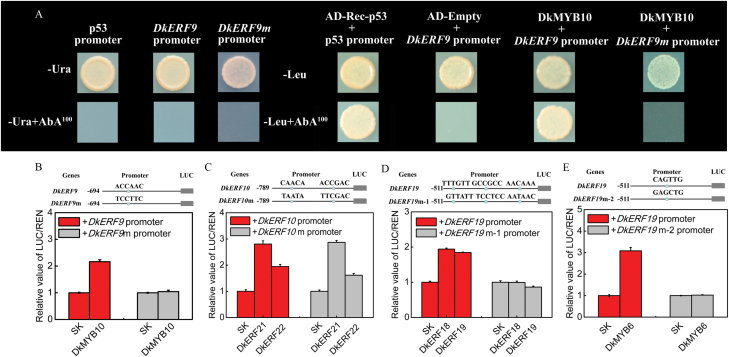
Analysis of interactions between TFs and promoters determined by (A) yeast one-hybrid and (B–E) motif mutation assays. (A) Yeast one-hybrid analysis. Auto-activation of promoters was tested on synthetically defined (SD) medium lacking Ura in the presence of aureobasidin A (–Ura+AbA^100^). Interactions were determined on SD medium lacking Leu in the presence of aureobasidin A (–Leu+AbA^100^). Positive control: AD-Rec-p53+p53 promoter, provided with the kit; negative control: AD-Empty+*DkERF9* promoter. (B–E) Schematic diagrams of motif mutations for the *DkERF9/10/19* promoters and results of dual-luciferase assays performed with original and mutated promoters. The ratio of LUC/REN of the empty vector (SK) plus promoter was used as the calibrator (set as 1). Values are means (+SE) from five replicates.

Using the yeast one-hybrid assay, it was found that DkMYB10 could physically bind to the *DkERF9* promoter (Fig. 4A). Furthermore, the MBSII (ACCAAC; Grotewold *et al.*, 1994) mutation in the *DkERF9* promoter abolished the effects of DkMYB10 (Fig. 4A, B). Since the *DkERF10* and *DkERF19* promoters auto-activated in yeast (see Supplementary Fig. S7), a combination of *cis*-element mutations and dual-luciferase assays was used as an alternative way to test the specificity of this interaction. For the *DkERF10* promoter, two motifs (CAACA, Kagaya *et al.*, 1999; ACCGAC, DRE element, Stockinger *et al.*, 1997) were mutated to TAATA and TTCGAC, respectively (Fig. 4C). Subsequent dual-luciferase assays indicated that DkERF21 and DkERF22 had similar activation on the *DkERF10* promoter or the mutated *DkERF10* promoter (*DkERF10* m-promoter), suggesting either the absence of a direct interaction or that the interaction occurs with other unknown *cis*-elements (Fig. 4C). Two different mutations were designed to test the interaction between three transcription factors (DkERF18, 19, and DkMYB6) and the promoter of *DkERF19*. Three motifs (TTTGTT/AACAAA, TTTGTT, Dinh *et al.*, 2012; GCCGCC, GCC box, Ohme-Takagi and Shinshi, 1995) were mutated to GTTATT/AATAAC and TCCTCC, and designated as *DkERF19m-1* (Fig. 4D). To test the DkMYB6 interaction, the CAGTTG motif (MBSI; Solano *et al.*, 1997) in the *DkERF19* promoter was mutated to GAGCTG, designated as *DkERF19m-2* (Fig. 4E). Dual-luciferase assays indicated that these motif mutations abolished trans-activation of the *DkERF19* promoter by DkERF18, 19, and DkMYB6 (Fig. 4D, E).

### Transient overexpression analysis in persimmon fruit discs

**Fig. 5.**
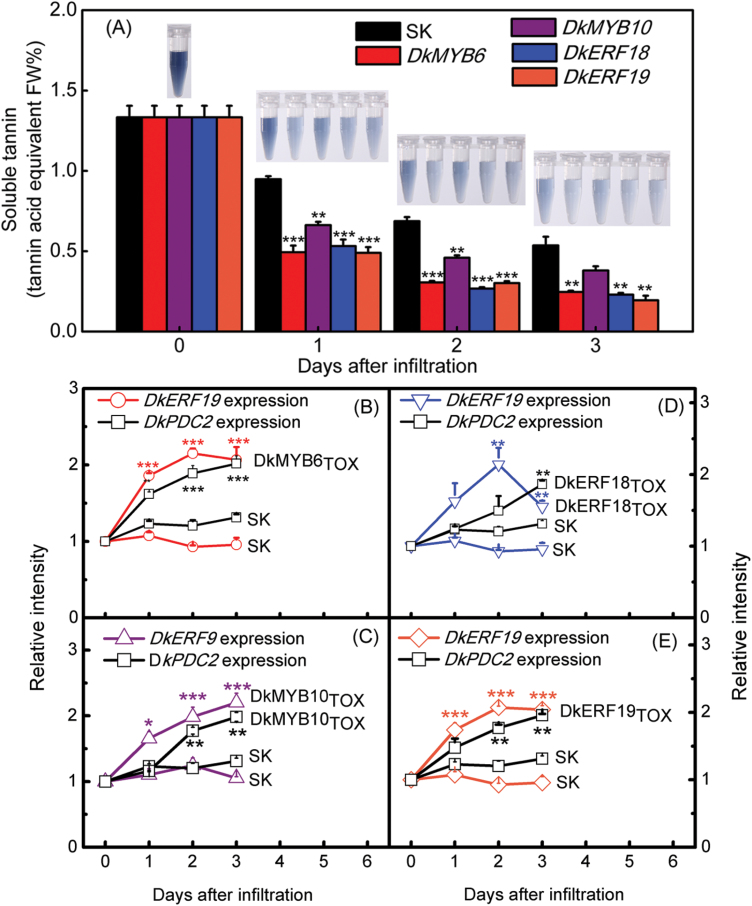
Transient over-expression of TFs in persimmon fruit discs. The transient over-expression experiments were conducted with the empty vector pGreen II 002962-SK (SK) and DkMYB6/10 and DkERF18/19. Tissues from each of the infiltrated discs were taken to measure the content of soluble tannins (A) and the relative gene expression levels of related downstream genes compared with the SK control (B–E) during the 3 d of infiltration. Soluble tannin contents were measured using Folin–Ciocalteu reagent and were quantitated as tannin acid equivalents. The images above the bars in (A) show the reaction liquids used for measuring the soluble tannin content: the darker the colour of the test solution the higher the content of soluble tannin. Values are means (±SE) from three biological replicates. (*P<0.05, **P<0.01, ***P<0.001).

Due to the difficulty of stable transformation of perennial fruit such as persimmon, transient overexpression (TOX) analyses were performed with fruit discs. *DkMYB6*, *DkMYB10*, *DkERF18*, and *DkERF19* were selected for analysis in view of their direct trans-activation of the *DkERF9* and *DkERF19* promoters (Figs 3 and 4), using tannin removal as a reporter for activity. The content of soluble tannins in the discs treated with transcription factors and the empty vector (SK, control) all declined during the incubation, which may have been due to the experimental manipulation (Fig. 5A). All four transcription factors, however, accelerated insolubilization of tannins from 1 d to 3 d, resulting in significantly lower content of soluble tannins than the controls (Fig. 5A). Interactions between transcription factors were also analysed and the results indicated that TOX of *DkMYB6* and *10* and *DkERF18* and *19* could significantly up-regulate the endogenous *DkERF9* or *DkERF19* transcripts in persimmon fruit discs, which further supported the interactions of AHCA-responsive transcription factors with the *ERF* promoters (Fig.5B–E). The expressions of downstream structure genes related deastringency were also analysed, and their expressions were also significantly up-regulated in the fruit discs, indicating that the transcriptional regulatory cascade would ultimately result in the regulation of structural genes (such as *DkADH1* and *DkPDC2*) and hence in regulation of fruit deastringency.

## Discussion

### Multiple TFs associated with the high-CO_2_/hypoxia response that leads to deastringency

AHCA treatment is the most effective commercial method for reducing persimmon fruit astringency. It functions by stimulating the accumulation of anaerobic metabolites, such as acetaldehyde, which precipitate soluble tannins (Supplementary Fig. S1; Pesis and Ben-Arie, 1984; Taira *et al.*, 1992; Arnal and Del Río, 2004), the loss of which acts as a unique reporter of the anaerobic response. Previous work has highlighted the role of *DkERF9*,*10*,*19*, and *22* in this process (Min *et al.*, 2012, 2014). In this present study, eight new TFs belonging to different families, including *MYB*, *bHLH*, *Zinc finger*, *HSF*, and *IAA*, were characterized and their transcripts shown to increase in abundance in response to AHCA treatment in three different cultivars, suggesting that multiple TFs may contribute to the deastringency process. These results are similar to those from omics-based analyses in Arabidopsis, where, in different organs and under different conditions, Liu *et al.* (2005) found 64 differentially expressed TFs in Arabidopsis seeds under hypoxic conditions, and Licausi *et al.* (2011*b*) identified over 180 TF genes, most of which belonged to the *ERF*, *bHLH*, *MYB*, *HSF*, and *Zinc finger* families, that were up- or down-regulated in roots under hypoxic conditions.

The *ERF*s are the best-characterized transcription factor gene family involved in plant hypoxia responses, and members belonging to Group VII play a key role (Hinz *et al.*, 2010; Licausi *et al.*, 2010, 2011*a*; Gibbs *et al.*, 2011; Yang *et al.*, 2011; Min *et al.*, 2012; Gasch *et al.*, 2016). The ERFs detected in this study and earlier research (Min *et al.*, 2014) in persimmon belong to Group VII (*DkERF10*), Group IV (the DREB family) (*DkERF9*), Group IX (*DkERF18*, *19*), and Group X (*DkERF21*, *22*). The DkERF10 protein, the only persimmon Group-VII ERF detected, is assumed to be stabilized due to the MC domain (MCGGAII), which contributes to the stability of hypoxia-responsive ERFs (Gibbs *et al.*, 2011; Licausi *et al.*, 2011*a*), but there is also a major increase in *DkERF10* mRNA on day 1 of anoxia (Min *et al.*, 2012). The other *DkERF* genes involved in the response lack this MC domain, but our results indicate that they nevertheless participate directly in the regulatory cascade. In Arabidopsis and other plants, multiple groups of *ERF*s have also been shown to be associated with the responses to hypoxic treatments (Licausi *et al.*, 2011*b*; Cukrov *et al.*, 2016). Moreover, all these eight AHCA-responsive TFs were not homologous to the known ‘core 49’ hypoxia-responsive genes as identified by Mustroph *et al.* (2009). Although the expression of MYBs and many other transcription factors has been correlated with hypoxia tolerance (Hoeren *et al.*, 1998; Abe *et al.*, 2003; Mattana *et al.*, 2007; Mustroph *et al.*, 2009; Fang *et al.*, 2016), it is worth emphasizing that among the eight AHCA-responsive TFs, only five genes were characterized as hypoxia-responsive with AHNA treatment, and the other three (*DkGT3-1*, *DkMYB10*, and *DkAN1-1*) were only responsive to high-CO_2_, and are thus not hypoxia-responsive (Supplementary Fig. S4). These findings indicated the similarities and also the differences between AHCA treatment in persimmon and hypoxia responses in model plants.

### Transcriptional regulatory cascade of AHCA-responsive TFs

**Fig. 6.**
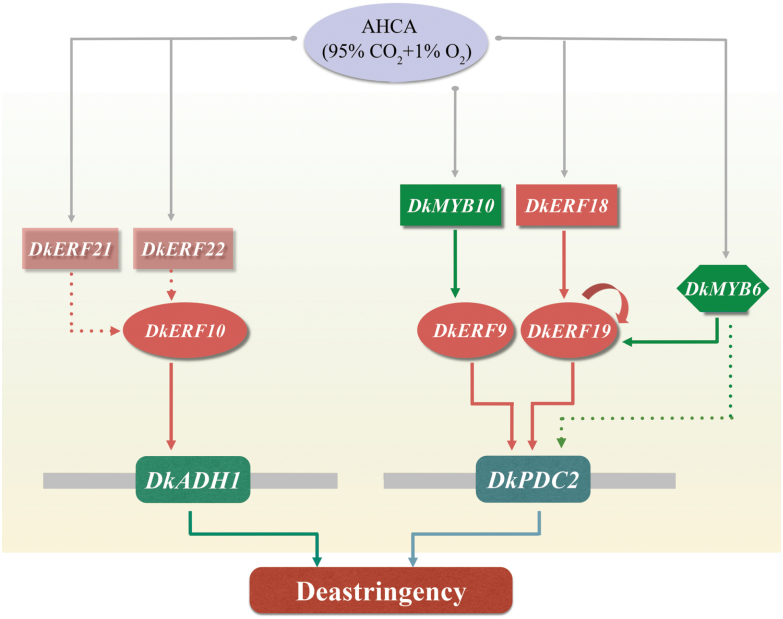
Model of TF interactions in response to artificial high-CO_2_ atmosphere (AHCA). AHCA treatment triggers the expression of various TFs, but only those with confirmed interactions are shown here. DkERF9, 10, and19 bind directly to, and activate promoters of, the anoxia-related genes *DkADH1* and *DkPDC2*, which subsequently accelerate deastringency. DkERF18, 19, and DkMYB6, 10 physically bind and trans-activate *DkERF* promoters; DkERF21 and 22 are indirect regulators of *DkERF10*. DkERF19 exhibits auto-activation and binds to its own promoter. Solid arrows indicate direct interactions, while dashed arrows indicate indirect regulation.

Although the mRNAs for *DkbHLH1*, *DkMYB9*,*10*,*11*, *DkRH2-1*, *DkGT3-1*, *DkAN1-1*, and *DkHSF1* increased in abundance in response to AHCA, the corresponding proteins did not have a significant direct trans-activation effect on the *DkADH1* and *DkPDC2* promoters (all responses being significantly below 2-fold) (Fig. 2). This suggested that the newly identified factors might function indirectly in stimulating deastringency, and a further investigation was conducted to test possible interactions between the AHCA-responsive TFs and *DkERF9*,*10*, and *19*, which recognize and trans-activate the *DkADH1* or *DkPDC2* promoters. The results indicated that there are at least two main types of transcriptional interactions between TFs: MYB–ERF and ERF–ERF interactions (Fig. 6). At least two MYBs, DkMYB6 and DkMYB10, physically bound to, and were putative activators of, the *DkERF19* and *DkERF9* promoters, respectively. ERF–ERF interactions included an indirect effect of DkERF21 and DkERF22 on the *DkERF10* promoter and a direct regulation by DkERF18 and DkERF19 of the *DkERF19* promoter (Fig. 6). *DkERF19* showed auto-activation in dual-luciferase assays, indicating that its own protein can bind and trans-activate its promoter, and as our knowledge of the TF cascade expands it will be important to test for similar interactions and auto-regulations between specific TFs (e.g. MYB and ERF) that may contribute to regulation of the high-CO_2_/hypoxia response (Fig. 6). It also worth highlighting *DkbHLH1*, which was significantly up-regulated by deastringency treatments in all three examined cultivars and had a limited (less than 2-fold, but nonetheless significant) effect on the *DkERF19* promoter. Compared to the TFs considered above, the regulatory mechanisms of *DkbHLH1* (as well as the other responsive TFs) in the response of fruit to hypoxia require further investigation.

Regulatory cascades between TFs have been widely reported in various plants; for example, *AtSND1*, a NAC transcription factor, is involved in the regulation of secondary wall biosynthesis in Arabidopsis through trans-activation of *AtMYB46* (Zhong *et al.*, 2007), and the MdMYB10 protein can bind and transactivate *MdMYB10*, which is involved in anthocyanin production in red-fleshed apples (Espley *et al.*, 2009). For hypoxia responses, some TFs have been characterized at the transcript level and correlated with the expression of hypoxia-responsive genes (e.g. *ADH* and *PDC*, Abe *et al.*, 2003; Licausi *et al.*, 2011*b*). Our TF–promoter interaction results showed that, although Group-VII ERFs may play a leading role in sensing anaerobic conditions, there is a transcriptional cascade that leads to the up-regulation of the fermentation genes *DkADH1* and *DkPDC2* that involves the Group-VII DkERF10, and DkERFs from other Groups (DkERF9,18,19,21,22) and DkMYB6 and 10 (Fig. 6). The model presented here is supported by physical-binding and trans-activation studies (Figs 3 and 4; Min *et al.*, 2012, 2014; Zhu *et al.*, 2016) that provide insight into a hierarchy of interactions between the components of a regulatory cascade, leading to anaerobic responses. It enhances our understanding of the mechanism of the fruit hypoxia response, and may also apply to similar responses in other plant organs. One mechanism for the action of DkERF10 is that the protein may be stabilized by the effect of low O_2_ on the MC domain. No TFs were found that could directly regulate the *DkERF10* promoter (Figs 3 and 4), although there was an indirect enhancement by DkERF21 and DkERF22. The possibility that there may be another unknown TF that can directly regulate the *DkERF10* promoter and/or that an unknown *cis*-element exists in the *DkERF10* promoter to which ERFs can bind requires further investigation.

### *In vivo* interactions between high-CO_2_/hypoxia-responsive TFs and their roles in insolublization of tannins in persimmon fruit

TOX analyses showed that *DkMYB6*_*TOX*_ and *10*_*TOX*_ and *DkERF18*_*TOX*_ and *19*_*TOX*_ could significantly accelerate insolubilization of tannins in persimmon fruit discs, indicating that they participate in causing deastringency, which results directly from anaerobiosis (Taira *et al.*, 2001; Salvador *et al.*, 2007). The advantage of the TOX system is that it allows the analysis of the regulation of the endogenous genes and of the role of their transcriptional regulators and tannin content. Examples of the successful use of TOX include overexpression of *DkMYB4* in kiwifruit calluses, which significantly enhanced tannin biosynthesis (Akagi *et al.*, 2009), and expression of *DkPDC2* in persimmon leaves, which decreased soluble tannin content (Min *et al.*, 2012). The expression of *DkERF9*, *DkERF19*, and *DkPDC2* was up-regulated by *DkMYB6*_*TOX*_ and *10*_*TOX*_ and *DkERF18*_*TOX*_ and *19*_*TOX*_ in discs over a 1–3 d period, indicating the rapid and continuous responses of endogenous genes to these TFs, which occurred concomitantly with the decrease in soluble tannins in fruit discs. These *in vitro* results (Fig. 5) confirm the potential interactions and roles for *ERF* and *MYB* TFs in the response of persimmon fruit to AHCA treatment (Fig. 6).

## Supplementary data

Supplementary data are available at *JXB* online.

Fig. S1. Effects of AHCA treatment on post-harvest deastringency in fruit of persimmon ‘Mopanshi’ at 20 °C.

Fig. S2. Expression of transcription factors that were relatively less responsive to AHCA treatment.

Fig. S3. Comparison of tannin printing assays for control and AHNA- and AHCA-treated ‘Gong cheng-shui shi’ fruit at 1 d.

Fig. S4. Expression of transcription factors in response to AHCA and AHNA treatment in ‘Gong cheng-shui shi’ fruit.

Fig. S5. Synergistic trans-activation effects of combinations of DkERF21 and DkERF22 on the *DkERF10* promoter.

Fig. S6. Synergistic trans-activation effects of combinations of DkMYB6 and DkERF18/19 on the *DkERF19* promoter.

Fig. S7. Auto-activation test for the *DkERF10/19* promoters.

Table S1. Primer sequences for 3′-RACE analysis.

Table S2. Primer sequences for 5′-RACE analysis.

Table S3. Primer sequences for full-length TFs.

Table S4. Primer sequences for real-time PCR analysis.

Table S5. Primer sequences for the dual-luciferase assays.

Table S6. Primer sequences for the yeast one-hybrid assay.

Table S7. Primer sequences for site-directed mutagenesis of the *DkERF9/10/19* promoters.

Table S8. Summary on hypoxia-responsive transcription factors from persimmon fruit.

Supplementary Figures Tables

## Abbreviations

ADH: alcohol dehydrogenase
AHCA: artificial high-CO_2_ atmosphere (1% O_2_ and 95% CO_2_)
AHNA: artificial high-N_2_ atmosphere (99% N_2_ and 1% O_2_)
PDC: pyruvate decarboxylase
ERF: ethylene-response factor
HRE: hypoxia-response ERF
TOX: transient overexpression.

## References

[CIT0001] Abe H, Urao T, Ito T, Seki M, Shinozaki K, Yamaguchi-Shinozaki K 2003 Arabidopsis AtMYC2 (bHLH) and AtMYB2 (MYB) function as transcriptional activators in abscisic acid signaling. The Plant Cell 15, 63–78. 12509522 10.1105/tpc.006130 PMC143451

[CIT0002] Akagi T, Ikegami A, Tsujimoto T, Kobayashi S, Sato A, Kono A, Yonemori K 2009 DkMyb4 is a Myb transcription factor involved in proanthocyanidin biosynthesis in persimmon fruit. Plant Physiology 151, 2028–2045. 19783643 10.1104/pp.109.146985 PMC2785967

[CIT0003] Ali S, Khan AS, Malik AU, Shahid M 2016 Effect of controlled atmosphere storage on pericarp browning, bioactive compounds and antioxidant enzymes of litchi fruits. Food Chemistry 206, 18–29. 27041293 10.1016/j.foodchem.2016.03.021

[CIT0004] Arnal L, Del Río MA 2004 Effect of cold storage and removal astringency on quality of persimmon fruit (*Diospyros kaki*, L.) cv. Rojo Brillante. Food Science & Technology International 10, 179–185.

[CIT0005] Banti V, Mafessoni F, Loreti E, Alpi A, Perata P 2010 The heat-inducible transcription factor *HsfA2* enhances anoxia tolerance in Arabidopsis. Plant Physiology 152, 1471–1483. 20089772 10.1104/pp.109.149815 PMC2832282

[CIT0006] Bekele EA, Alis ARR, Hertog MLATM, Nicolai BM, Geeraerd AH 2016 Dynamics of metabolic adaptation during initiation of controlled atmosphere storage of ‘Jonagold’ apple: effects of storage gas concentrations and conditioning. Postharvest Biology and Technology 117, 9–20.

[CIT0007] Branco-Price C, Kawaguchi R, Ferreira RB, Bailey-Serres J 2005 Genome-wide analysis of transcript abundance and translation in Arabidopsis seedlings subjected to oxygen deprivation. Annals of Botany 96, 647–660. 16081496 10.1093/aob/mci217 PMC4247032

[CIT0008] Bui LT, Giuntoli B, Kosmacz M, Parlanti S, Licausi F 2015 Constitutively expressed ERF-VII transcription factors redundantly activate the core anaerobic response in *Arabidopsis thaliana*. Plant Science 236, 37–43. 26025519 10.1016/j.plantsci.2015.03.008

[CIT0009] Chang S, Puryear J, Cairney J 1993 A simple and efficient method for isolating RNA from pine trees. Plant Molecular Biology Reporter 11, 113–116.

[CIT0010] Cukrov D, Zermiani M, Brizzolara S et al 2016 Extreme hypoxic conditions induce selective molecular responses and metabolic reset in detached apple fruit. Frontiers in Plant Science 7, 146. 26909091 10.3389/fpls.2016.00146 PMC4754620

[CIT0011] Dinh TT, Girke T, Liu X, Yant L, Schmid M, Chen X 2012 The floral homeotic protein APETALA2 recognizes and acts through an AT-rich sequence element. Development 139, 1978–1986. 22513376 10.1242/dev.077073 PMC3347690

[CIT0012] Espley RV, Brendolise C, Chagné D et al 2009 Multiple repeats of a promoter segment causes transcription factor autoregulation in red apples. The Plant Cell 21, 168–183. 19151225 10.1105/tpc.108.059329 PMC2648084

[CIT0013] Fang F, Wang MM, Zhu QG, Min T, Grierson D, Yin XR, Chen KS 2016 *DkMYB6* is involved in persimmon fruit deastringency, via transcriptional activation on both *DkPDC* and *DkERF*. Postharvest Biology and Technology 111, 161–167.

[CIT0014] Gasch P, Fundinger M, Müller JT, Lee T, Bailey-Serres J, Mustroph A 2016 Redundant ERF-VII transcription factors bind to an evolutionarily conserved *cis*-motif to regulate hypoxia-responsive gene expression in arabidopsis. The Plant Cell 28, 160–180. 26668304 10.1105/tpc.15.00866 PMC4746684

[CIT0015] Gibbs DJ, Lee SC, Isa NM et al 2011 Homeostatic response to hypoxia is regulated by the N-end rule pathway in plants. Nature 479, 415–418. 22020279 10.1038/nature10534 PMC3223408

[CIT0016] Grotewold E, Drummond BJ, Bowen B, Peterson T 1994 The *myb*-homologous *P* gene controls phlobaphene pigmentation in maize floral organs by directly activating a flavonoid biosynthetic gene subset. Cell 76, 543–553. 8313474 10.1016/0092-8674(94)90117-1

[CIT0017] Hellens RP, Allan AC, Friel EN, Bolitho K, Grafton K, Templeton MD, Karunairetnam S, Gleave AP, Laing WA 2005 Transient expression vectors for functional genomics, quantification of promoter activity and RNA silencing in plants. Plant Methods 1, 13. 16359558 10.1186/1746-4811-1-13 PMC1334188

[CIT0018] Hinz M, Wilson IW, Yang J, Buerstenbinder K, Llewellyn D, Dennis ES, Sauter M, Dolferus R 2010 Arabidopsis *RAP2.2*: an ethylene response transcription factor that is important for hypoxia survival. Plant Physiology 153, 757–772. 20357136 10.1104/pp.110.155077 PMC2879770

[CIT0019] Hoeren FU, Dolferus R, Wu Y, Peacock WJ, Dennis ES 1998 Evidence for a role for AtMYB2 in the induction of the Arabidopsis alcohol dehydrogenase gene (*ADH1*) by low oxygen. Genetics 149, 479–490. 9611167 10.1093/genetics/149.2.479 PMC1460183

[CIT0020] Ikegami A, Eguchi S, Kitajima A, Inoue K, Yonemori K 2007 Identification of genes involved in proanthocyanidin biosynthesis of persimmon (*Diospyros kaki*) fruit. Plant Science 172, 1037–1047.

[CIT0021] Kagaya Y, Ohmiya K, Hattori T 1999 RAV1, a novel DNA-binding protein, binds to bipartite recognition sequence through two distinct DNA-binding domains uniquely found in higher plants. Nucleic Acids Research 27, 470–478. 9862967 10.1093/nar/27.2.470 PMC148202

[CIT0022] Kennedy RA, Rumpho ME, Fox TC 1992 Anaerobic metabolism in plants. Plant Physiology 100, 1–6. 16652929 10.1104/pp.100.1.1 PMC1075508

[CIT0023] Lasanthi-Kudahettige R, Magneschi L, Loreti E, Gonzali S, Licausi F, Novi G, Beretta O, Vitulli F, Alpi A, Perata P 2007 Transcript profiling of the anoxic rice coleoptile. Plant Physiology 144, 218–231. 17369434 10.1104/pp.106.093997 PMC1913783

[CIT0024] Lee SC, Mustroph A, Sasidharan R, Vashisht D, Pedersen O, Oosumi T, Voesenek LA, Bailey-Serres J 2011 Molecular characterization of the submergence response of the *Arabidopsis thaliana* ecotype Columbia. New Phytologist 190, 457–471. 21231933 10.1111/j.1469-8137.2010.03590.x

[CIT0025] Lee TG, Jang CS, Kim JY, Kim DS, Park JH, Kim DY, Seo YW 2007 A Myb transcription factor (*TaMyb1*) from wheat roots is expressed during hypoxia: roles in response to the oxygen concentration in root environment and abiotic stresses. Physiology Plantarum 129, 375–385.

[CIT0026] Licausi F, Kosmacz M, Weits DA, Giuntoli B, Giorgi FM, Voesenek LA, Perata P, van Dongen JT 2011a Oxygen sensing in plants is mediated by an N-end rule pathway for protein destabilization. Nature 479, 419–422. 22020282 10.1038/nature10536

[CIT0027] Licausi F, van Dongen JT, Giuntoli B, Novi G, Santaniello A, Geigenberger P, Perata P 2010 *HRE1* and *HRE2*, two hypoxia-inducible ethylene response factors, affect anaerobic responses in *Arabidopsis thaliana*. The Plant Journal 62, 302–315. 20113439 10.1111/j.1365-313X.2010.04149.x

[CIT0028] Licausi F, Weits DA, Pant BD, Scheible WR, Geigenberger P, van Dongen JT 2011b Hypoxia responsive gene expression is mediated by various subsets of transcription factors and miRNAs that are determined by the actual oxygen availability. New Phytologist 190, 442–456. 20840511 10.1111/j.1469-8137.2010.03451.x

[CIT0029] Liu F, Vantoai T, Moy LP, Bock G, Linford LD, Quackenbush J 2005 Global transcription profiling reveals comprehensive insights into hypoxic response in Arabidopsis. Plant Physiology 137, 1115–1129. 15734912 10.1104/pp.104.055475 PMC1065411

[CIT0030] Livak KJ, Schmittgen TD 2001 Analysis of relative gene expression data using real-time quantitative PCR and the 2^–ΔΔ*C*^T method. Methods 25, 402–408. 11846609 10.1006/meth.2001.1262

[CIT0031] Matityahu I, Marciano P, Holland D, Ben-Arie R, Amir R 2016 Differential effects of regular and controlled atmosphere storage on the quality of three cultivars of pomegranate (*Punica granatum* L.). Postharvest Biology and Technology 115, 132–141.

[CIT0032] Mattana M, Vannini C, Espen L et al 2007 The rice Mybleu transcription factor increases tolerance to oxygen deprivation in Arabidopsis plants. Physiologia Plantarum 131, 106–121. 18251929 10.1111/j.1399-3054.2007.00936.x

[CIT0033] Min T, Fang F, Ge H, Shi YN, Luo ZR, Yao YC, Grierson D, Yin XR, Chen KS 2014 Two novel anoxia-induced ethylene response factors that interact with promoters of deastringency-related genes from persimmon. PLoS ONE 9, e97043. 24805136 10.1371/journal.pone.0097043 PMC4013125

[CIT0034] Min T, Wang MM, Wang H, Liu X, Fang F, Grierson D, Yin XR, Chen KS 2015 Isolation and expression of *NAC* genes during persimmon fruit postharvest astringency removal. International Journal of Molecular Sciences 16, 1894–1906. 25599529 10.3390/ijms16011894 PMC4307340

[CIT0035] Min T, Yin XR, Shi YN, Luo ZR, Yao YC, Grierson D, Ferguson IB, Chen KS 2012 Ethylene-responsive transcription factors interact with promoters of *ADH* and *PDC* involved in persimmon (*Diospyros kaki*) fruit de-astringency. Journal of Experimental Botany 63, 6393–6405. 23095993 10.1093/jxb/ers296 PMC3504493

[CIT0036] Mo RL, Yang SC, Huang YM, Chen WX, Zhang QL, Luo ZR 2016 *ADH* and *PDC* genes involved in tannins coagulation leading to natural de-astringency in Chinese pollination constant and non-astringency persimmon (*Diospyros kaki* Thunb.). Tree Genetics & Genomes 12, 17.

[CIT0037] Mustroph A, Zanetti ME, Jang CJH, Holtan HE, Repetti PP, Galbraith DW, Girke T, Bailey-Serres J 2009 Profiling translatomes of discrete cell populations resolves altered cellular priorities during hypoxia in *Arabidopsis*. Proceedings of the National Academy of Sciences, USA 106, 18843–18848. 10.1073/pnas.0906131106 PMC2764735 19843695

[CIT0038] Ohme-Takagi M, Shinshi H 1995 Ethylene-inducible DNA binding proteins that interact with an ethylene-responsive element. The Plant Cell 7, 173–182. 7756828 10.1105/tpc.7.2.173 PMC160773

[CIT0039] Papdi C, Pérez-Salamó I, Joseph MP, Giuntoli B, Bögre L, Koncz C, Szabados L 2015 The low oxygen, oxidative and osmotic stress responses synergistically act through the ethylene response factor VII genes *RAP2.12*, *RAP2.2* and *RAP2.3*. The Plant Journal 82, 772–784. 25847219 10.1111/tpj.12848

[CIT0040] Pesis E, Ben-Arie R 1984 Involvement of acetaldehyde and ethanol accumulation during induced deastringency of persimmon fruits. Journal of Food Science 49, 896–899.

[CIT0041] Salvador A, Arnal L, Besada C, Larrea V, Quiles A, Pérez-Munuera I 2007 Physiological and structural changes during ripening and deastringency treatment of persimmon fruit cv. ‘Rojo Brillante’. Postharvest Biology and Technology 46, 181–188.

[CIT0042] Sasidharan R, Mustroph A 2011 Plant oxygen sensing is mediated by the N-end rule pathway: a milestone in plant anaerobiosis. The Plant Cell 23, 4173–4183. 22207573 10.1105/tpc.111.093880 PMC3269858

[CIT0043] Solano R, Fuertes A, Sánchez-Pulido L, Valencia A, Paz-Ares J 1997 A single residue substitution causes a switch from the dual DNA binding specificity of plant transcription factor MYB.Ph3 to the animal c-MYB specificity. The Journal of Biological Chemistry 272, 2889–2895. 9006933 10.1074/jbc.272.5.2889

[CIT0044] Stockinger EJ, Gilmour SJ, Thomashow MF 1997 *Arabidopsis thaliana CBF1* encodes an AP2 domain-containing transcriptional activator that binds to the C-repeat/DRE, a cis-acting DNA regulatory element that stimulates transcription in response to low temperature and water deficit. Proceedings of the National Academy of Sciences, USA 3, 1035–1040. 10.1073/pnas.94.3.1035 PMC19635 9023378

[CIT0045] Taira S, Ikeda K, Ohkawa K 2001 Comparison of insolubility of tannins induced by acetaldehyde vapor in fruit of three types of astringent persimmon. Journal of the Japanese Society for Horticultural Science 48, 684–687.

[CIT0046] Taira S, Oba S, Watanabe S 1992 Removal of astringency from ‘Hiratanenashi’ persimmon fruit with a mixture of ethanol and carbon dioxide. Journal of the Japanese Society for Horticultural Science 61, 437–443.

[CIT0047] van Veen H, Akman M, Jamar DC, Vreugdenhil D, Kooiker M, van Tienderen P, Voesenek LA, Schranz ME, Sasidharan R 2014 Group VII ethylene response factor diversification and regulation in four species from flood-prone environments. Plant, Cell & Environment 37, 2421–2432. 10.1111/pce.12302 24548060

[CIT0048] Voesenek LA, Bailey-Serres J 2015 Flood adaptive traits and processes: an overview. New Phytologist 206, 57–73. 25580769 10.1111/nph.13209

[CIT0049] Xie XL, Yin XR, Chen KS 2016 Roles of APETALA2/Ethylene Responsive Factors in regulation of fruit quality. Critical Reviews in Plant Sciences 35, 120–130.

[CIT0050] Xu K, Xu X, Fukao T, Canlas P, Maghirang-Rodriguez R, Heuer S, Ismail AM, Bailey-Serres J, Ronald PC, Mackill DJ 2006 *Sub1A* is an ethylene-response-factor-like gene that confers submergence tolerance to rice. Nature 442, 705–708. 16900200 10.1038/nature04920

[CIT0051] Yang CY, Hsu FC, Li JP, Wang NN, Shih MC 2011 The AP2/ERF transcription factor AtERF73/HRE1 modulates ethylene responses during hypoxia in Arabidopsis. Plant Physiology 156, 202–212. 21398256 10.1104/pp.111.172486 PMC3091062

[CIT0052] Yin XR, Shi YN, Min T, Luo ZR, Yao YC, Xu Q, Ferguson I, Chen KS 2012 Expression of ethylene response genes during persimmon fruit astringency removal. Planta 235, 895–906. 22101946 10.1007/s00425-011-1553-2

[CIT0053] Zhong R, Richardson EA, Ye ZH 2007 The MYB46 transcription factor is a direct target of SND1 and regulates secondary wall biosynthesis in Arabidopsis. The Plant Cell 19, 2776–2792. 17890373 10.1105/tpc.107.053678 PMC2048704

[CIT0054] Zhu QG, Wang MM, Gong ZY, Fang F, Sun NJ, Li X, Grierson D, Yin XR, Chen KS 2016 Involvement of *DkTGA1* transcription factor in anaerobic response leading to persimmon fruit postharvest de-astringency. PLoS ONE 11, e0155916. 27196670 10.1371/journal.pone.0155916 PMC4873192

