# Supplementary material for: A transcription factor network responsive to high CO2/hypoxia is involved in deastringency in persimmon fruit
Source: J Exp Bot. 2018 Jan 30;69(8):2061–70. doi: 10.1093/jxb/ery028 (PMC6018754; doi:10.1093/jxb/ery028)
Supplement: Supplementary Figures Tables [file ery028_suppl_supplementary_figures_s1-s7_tables_s1-s8.pdf]

Figure S1

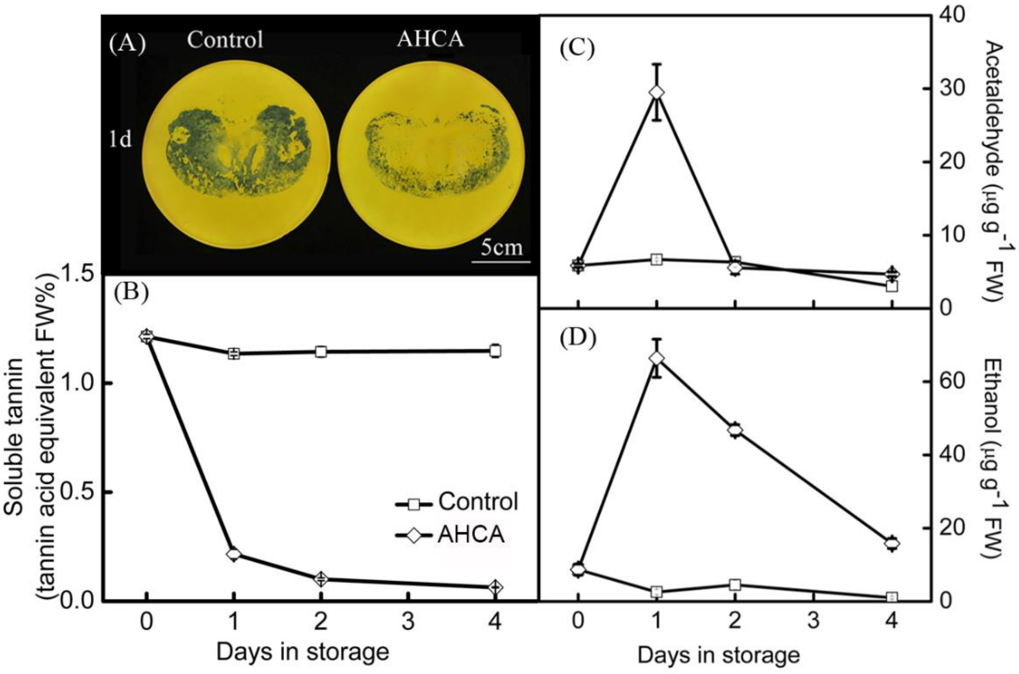

Figure S1 Effects of AHCA (95 % CO_2 + 1 % O_2, 1 d) treatment on 'Mopanshi' persimmon fruit postharvest deastringency at 20 °C

Figure S2

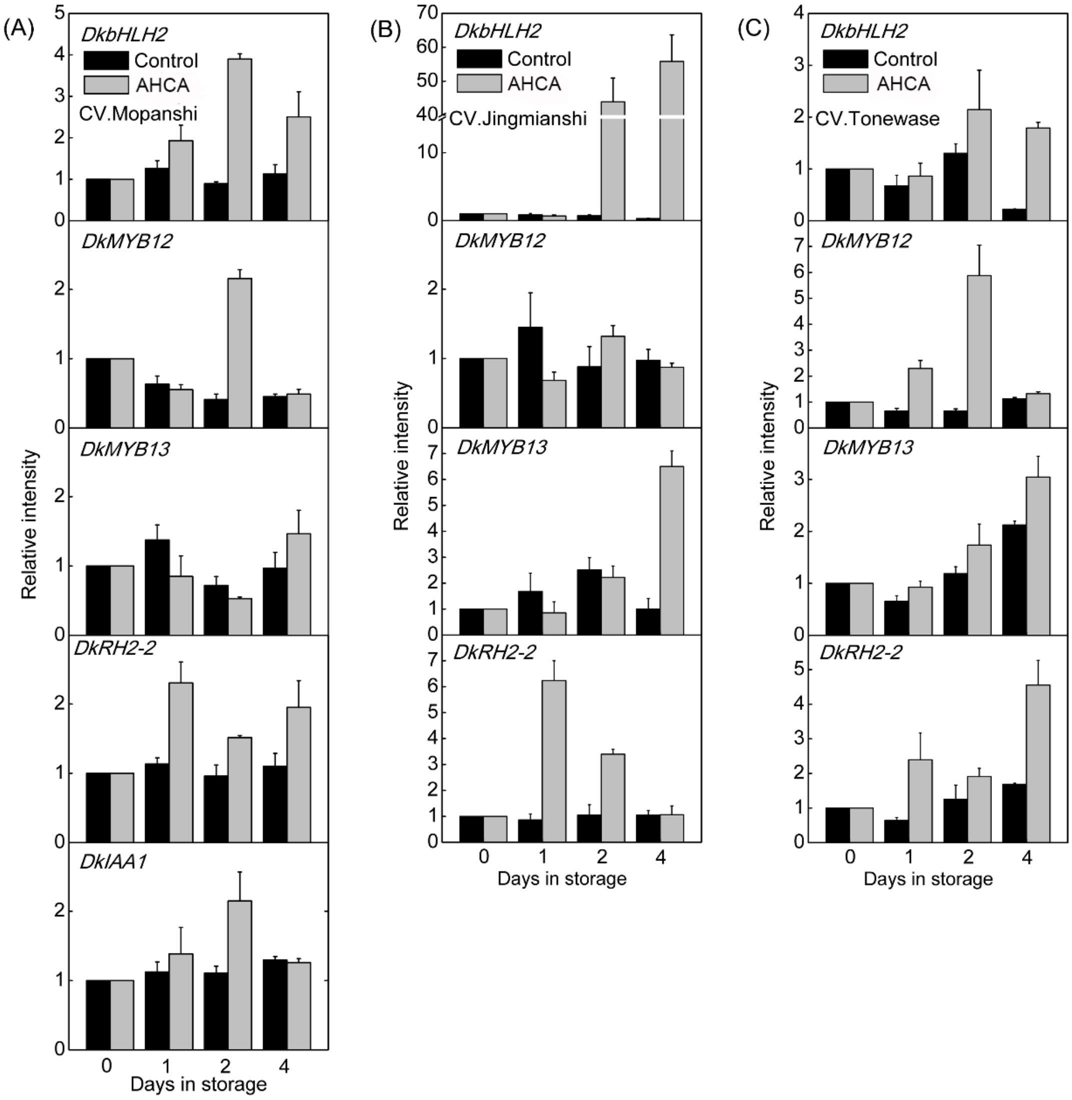

Figure S2. Expression of transcription factors were less responsive to AHCA treatment (*DkIAA1* did not express in 'Jingmianshi' and 'Tonewase')

Figure S3

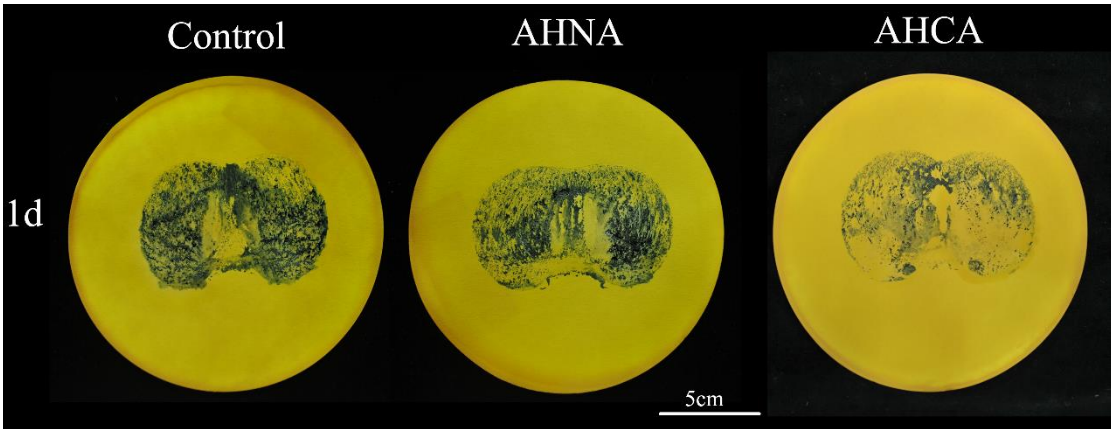

Figure S3. Comparison of tannin printing of control and AHNA and AHCA treaterd "Gong cheng-shui shi" fruit at 1 day in storage.

Figure S4

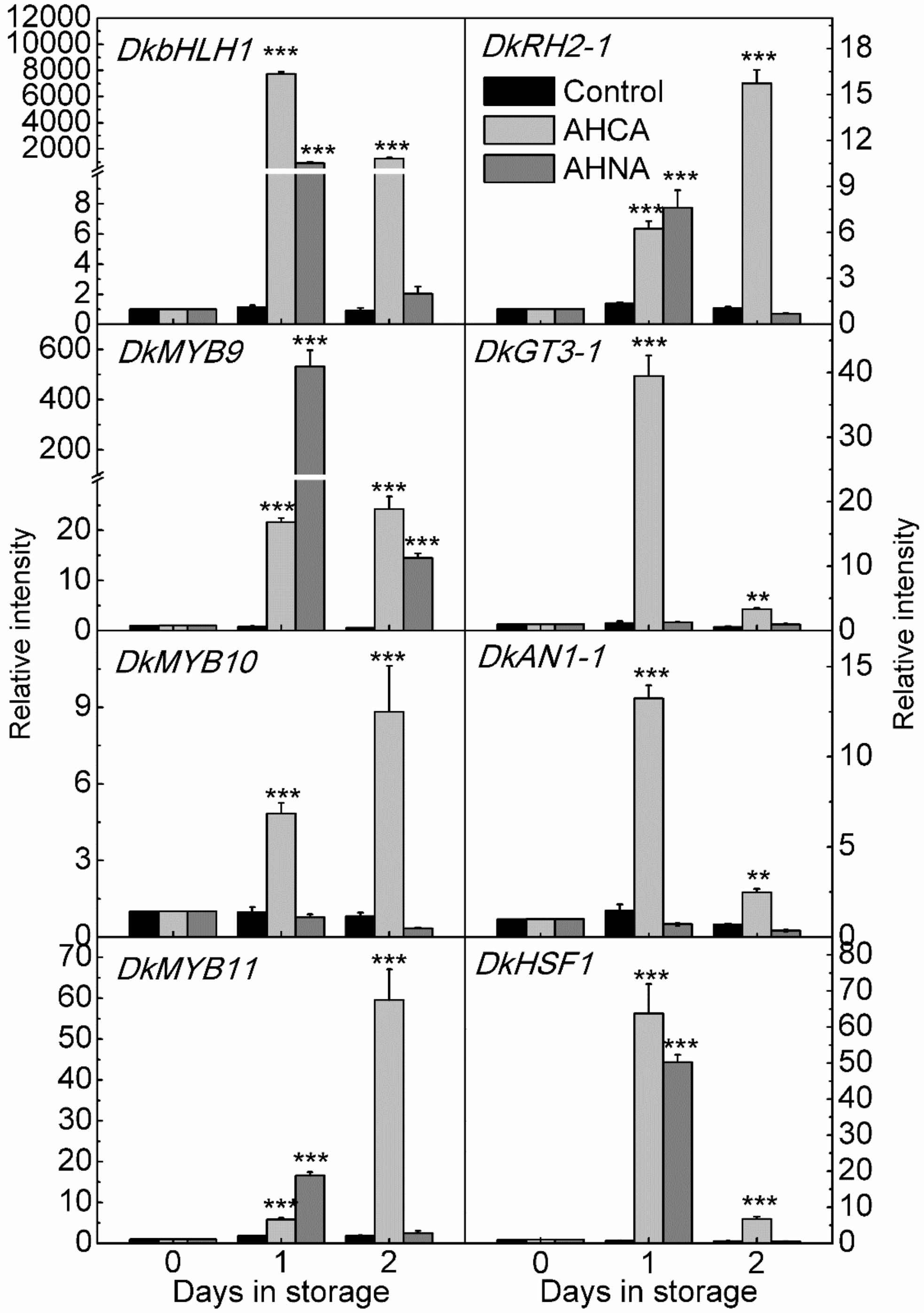

Figure S4. Expression of transcription factors were responsive to AHCA and AHNA treatment in "Gong cheng-shui shi"

Figure S5

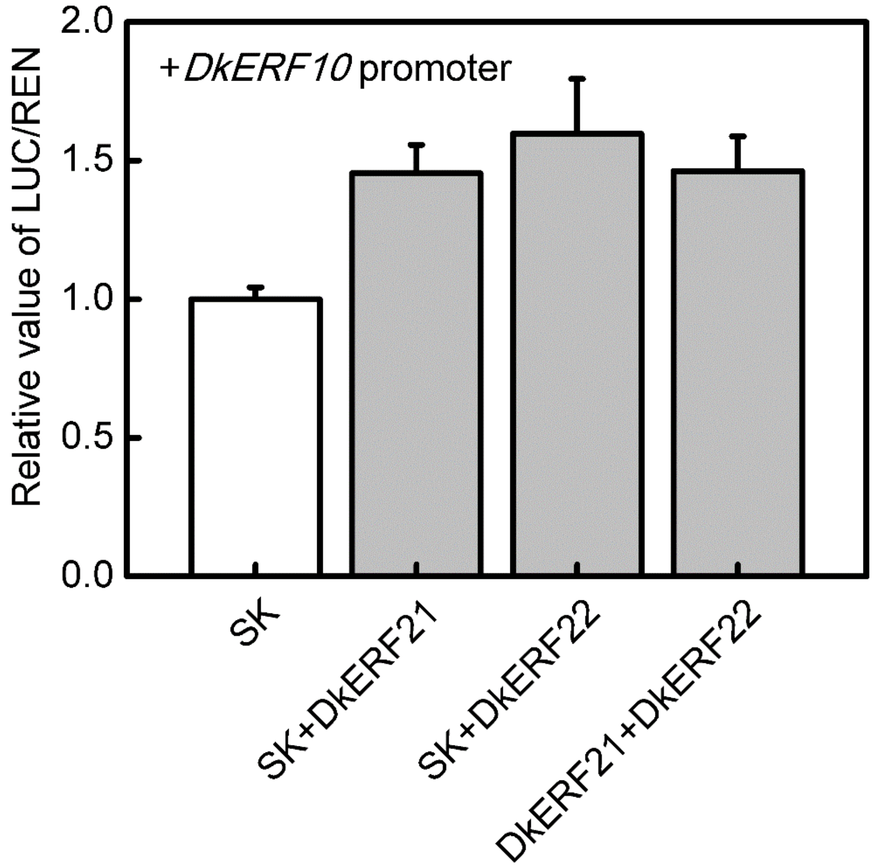

Figure S5. Synergistic trans-activation effect of combination of DkERF21 and DkERF22 on *DkERF10* promoter.

Figure S6

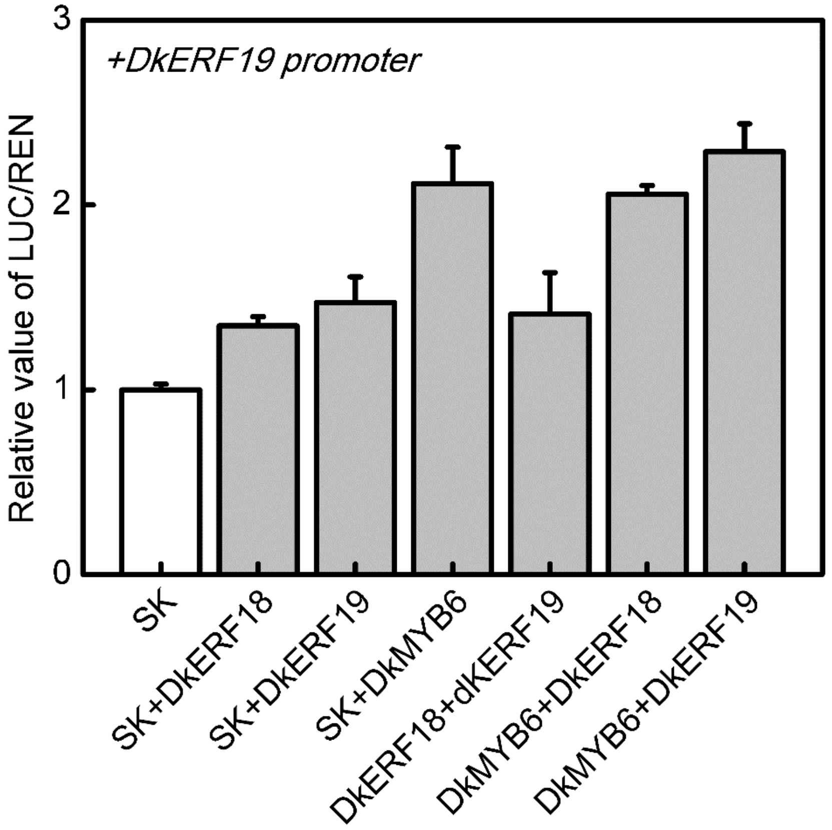

Figure S6. Synergistic trans-activation effect of combination of DkMYB6 and DkERF18/19 on *DkERF19* promoter.

Figure S7

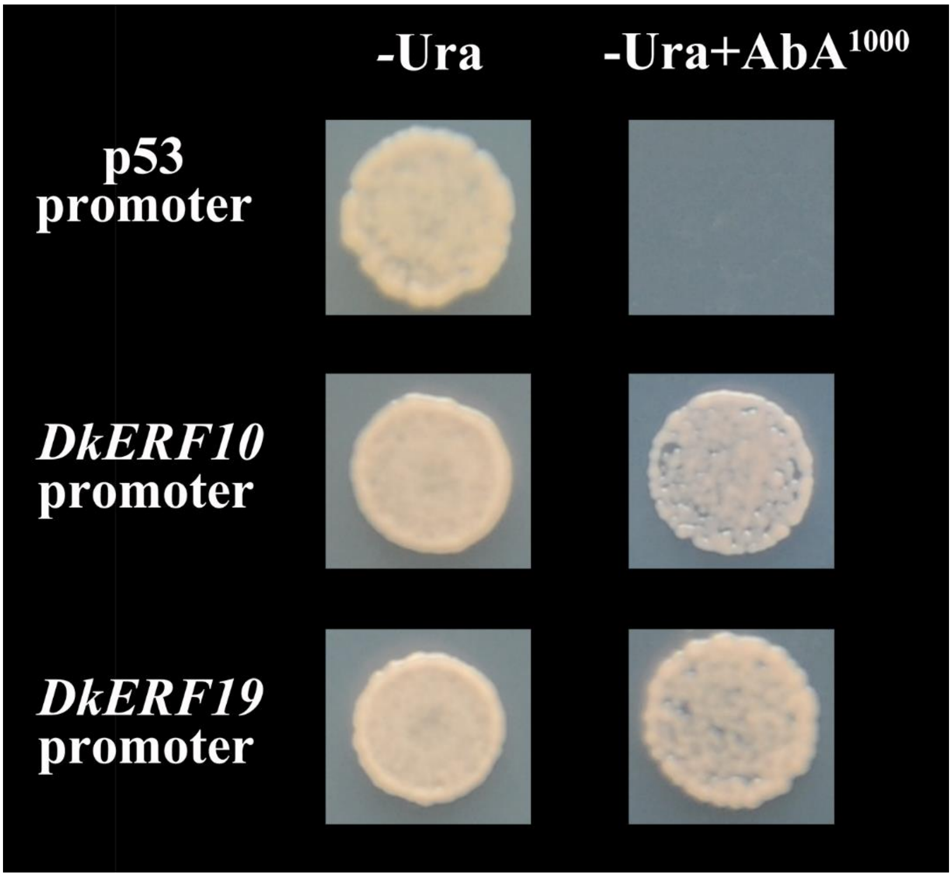

Figure S7. Auto-activation test for *DkERF10/19* promoters

Supplemental Table 1

Primer sequences for 3’-RACE analysis

Gene	Gene-specific primer (5’ to 3’)	Nested gene-specific primer (5’ to 3’)
DkMYB9	GACCATCATCAGCAATCCATTCGTCAAA	CAGAATCATCATGATCAGAACAATATTC
DkMYB10	CGCCACCTTCTTCTACTCCGGCCGCGGC	GGTGGATGGGCTTAGTAATGATGAAGTG
DkMYB11	GGTCTGTCATAGCTGCTCAACTACCAGG	CGGGCAACCATGAAGACAACTCATGGCG
DkMYB12	CGGCGTCGCCGCCGATCCAGCCTTTTCG	GCCGGCAAAGCTCATCCATCCTACTCCA
DkMYB13	GAACGGACAACGAGATCAAGAATTACTG	CCTGGAGCTGAGAATTAGCGCTCCACAC
DkbHLH1	CATGGCCAACAACGACCGAGTTCCAGGC	CTATCAGGAAGCTAGGATTAAGAGAGCG
DkbHLH2	CATCAGGCACCAGAGATCCAGTAAAGGC	GGCCTCTGCCGGCGAGGGCACATCTTCT
DkRH2-1	CCCGGGAGATGCAAAGAAGAAGATGAAG	GGTGGGTTGACCATGGCCAAGCCACTTG
DkRH2-2	GTTGTCGTCGAGCTGTGAACGGATTGCA	CCAGAATTCGCAACCACCTCTGTGGTTG
DkGT3-1	GCTATGGGTGGAAGCTGAAGGCGGAGCG	CAACAGTTTGAAGGAGAGTTTGGAGGAG
DkHSF1	CACTGCAAGTTCTCGGTGTCCACCCTCG	GCTTGGAAAGCGGATGCCTATGTCTATG
DkIAA1	CACCAGCTTCTGCCAATCCAGCTGTGCC	CGCTGTCCCAAGCAAGCCTCCCACTGAA
DkAN1-1	GTTGTGGTCTGCGAAATCTGCTCCACGG	GGCACGAGAAGTCCGACGTCTGCGATCC

Supplemental Table 2

Primer sequences for 5-RACE analysis

Gene	Gene-specific primer (5’ to 3’)	Nested gene-specific primer (5’ to 3’)
DkMYB9	GCGAAGCAAATGGGAATTTGACGAATGG	AACCGTCGCCTCCGCCAGTTTAGTGAGC
DkMYB10	CTTTCTTGAAGGGATGAAAAGCACCGCC	TTGTCATTGCTCTTGCCATCTTTATGAG
DkMYB11	TGCAGCTGCCATTTGCTTCTTCGTCTTC	GACAACTCTTGCCACAGCGCTTCAATCC
DkMYB12	GCTGGCAACTTGTGTTGGCGTGCGGGTC	GCGGCTGCAACGTCGTCGCCGTTGGAGG
DkMYB13	GCTCGTTCATCGGTCTGTGGGTTACCGG	GGGGAGGGAGCGCCAGCAGCCCTCGCCG
DkbHLH1	GGCTCTCCATGGACAGCGATCTGAGCG	GCTGGGCTTCGGCGGAGGCCAGACCTTG
DkbHLH2	TTCCAGCCTTTACTGGATCTCTGGTGCC	AGAAGCTTGCGAAGTTTTGAGCTCTTCC
DkRH2-1	TCTTCATCTTCTTCTTTGCATCTCCCGG	CTTCTCCTTGTCTCCTTCGAACAGTGGG
DkRH2-2	TGGTTAGAGCCACTAAGGCAAGCTATGG	CCTGGGTATCCACAAGGCTTGGGTTGGC
DkGT3-1	CGCTCTCCTCGTTGTCGTCGTCGTCGTC	CATTTGCATTTGCACTGCTCCGCGCTGC
DkHSF1	CGTTGACAAACTCCCATCTATCTGGATC	GGAGGGAAAGAATCAAGAAGCCCCTCCC
DkIAA1	GGCACAGCTGGATTGGCAGAAGCTGGTG	CCTGGTGGAGCAAGCCTCAACTCAAGGC
DkAN1-1	GCAGACGTCGGACTTCTCGTGCCTCTCC	GGACCTGTACGATCTGTGCTCCAAACAG

Supplemental Table 3

Primer sequences for full length TFs

Gene	Gene-specific primer (5’ to 3’)	Nested gene-specific primer (5’ to 3’)
DkMYB9	GGACGCCTGAAGAAGATCAGAC	CAGCTCTTTCACATTAATCCTTCC
DkMYB10	GGATCTCTCTATGCTCATACC	CTCTAATTAGTATCTGAACCGAAG
DkMYB11	GCAGCCATGGGAAGAGCTCCCTGC	GTAGGTAGTAGGTTCCTATGAAG
DkMYB12	CCTGATTCCTAGAAGAAGACCGCC	CAACTGATTACAACAAGACATCAC
DkMYB13	GGGCCTGCTGCTCAATTAAGC	CTCTCTCTCTCCTTGTTGCTAC
DkbHLH1	GGAGAGACAAAGAGAGAGAGGGAG	CATTGACCATAACCTGTCGCCGCC
DkbHLH2	GTAGCCTAACCACCAGTTTCAC	CTTCTTCCTGAATATTATGGAGC
DkRH2-1	CTAACGCCCACCATGTGAGAAG	CGAACACCCTCCTCCCTCAGAGG
DkRH2-2	GATGGCCACCCAAATTAATAAAAC	CGAAACTGTAAACCATCGATGGCC
DkGT3-1	GGTGGAGAATCTGATTCATATATC	GTTGCAAGACAAACCTCTCATGGC
DkHSF1	GGAGAAACCTGCTCATGAATATTG	CATTGTTGAACAGATTACTTATCCG
DkIAA1	GATCTGGGTGTTCACTAGGTTTTG	GAAACACGTCATACACAGTTGCTCC
DkAN1-1	CCTCATCCAGTTCAAGAAGCAAG	GAATCTCAGCAAACAATTTGTGCC

Supplemental Table 4

Table S4. Primer sequences for Real-time PCR analysis

Gene	Gene-specific primer (5' to 3')	Nested gene-specific primer (5' to 3')
DkMYB9	AGATTTGCCGGCATTAACAA	TCACACTACCAGCTCTTTCACA
DkMYB10	GTCTAGAGCAGCCGGAGCTA	CCGGAGAATTGGAACGAG
DkMYB11	GGGATTTGATATTAAGGGCATT	GGTCCAGACCCTAGCTTTCA
DkMYB12	TTGGCCTTGGTCATTTGTCT	CAAATGGAATGCCATGACTG
DkMYB13	TCAGAAGCTTGGAAATGAAATG	TCGATCCCGACAATCATACA
DkbHLH1	TTCACCATTTTCCCCCATTA	TCACTTGGATGGGATTTTCTG
DkbHLH2	CAGAAAGTGGTTTCGTGAAGA	TCTGGACACTGAAAGAAAGCAG
DkRH2-1	TCGATAAGTCCAACCAGCTCT	AACACCCTCCTCCCTCAGA
DkRH2-2	CGATCCGAATTTGGACTATGA	CCATACTCCCTTCTACGTATCACA
DkGT3-1	GCTAGGTACCTCAGTAGTGGTTG	TGCATGTGGTGTAATTAAGACG
DkHSF1	ACGTGAAGAAAGGGGAGCTT	CCATTCAATTCCCTCTTCCA
DkIAA1	AGCTCTCCTCGTTGTGCTGT	TTTGGTGGGAAAATGAGAGC
DkAN1-1	ATTTCTCCGCCCAATACTCC	CCAAATTGGTCTTGGTGTGA

Supplemental Table 5

Primer sequences for dual-luciferase assay experiment

Gene	Gene-specific primer (5’ to 3’)	Nested gene-specific primer (5’ to 3’)
DkMYB9	CGCGCGGCCGCATGTGTGTGTGTGTGTGT	GATACTAGTTCATTTATCGAGACGAGG
DkMYB10	CGCGCGGCCGCATGATCCGCAATACCCAC	GATACTAGTTCATGTGCTACGAGTAGA
DkMYB11	CGCGCGGCCGCATGGGAAGAGCTCCCTGC	GATACTAGTTCATATATAGAGCCCTCG
DkbHLH1	CGCGCGGCCGCATGGACTCCATCTTCCTG	GATACTAGTTCAGTGTGCCATGTCAGC
DkRH2-1	CGCGCGGCCGCATGTTGTCAAGATCATTT	GATACTAGTTCACTGGAAAAGGGAATC
DkGT3-1	CGCGCGGCCGCATGGAAGGAGGGCATCAC	GATACTAGTCTACAAGTCGTCTCTTCC
DkHSF1	CGCGCGGCCGCATGAATATTTCTCCAGGC	GATACTAGTTCACTCCCCTTCATTCAA
DkAN1-1	CGCGCGGCCGCATGGCAGGAACAGAGGCT	GATACTAGTTTAACATGCTTTGACAGA

Supplemental Table 6

Primer sequences for yeast experiment

Gene	Gene-specific primer (5' to 3')	Nested gene-specific primer (5' to 3')
DkMYB10	GCCATGGAGGCCAGTGAATTCATGGCATCAACATTCACGAG	CAGCTCGAGCTCGATGGATCCTCATGTGCTACGAGTAGAG

Supplemental Table 7

Primer sequences for site-directed mutagenesis of DkERF10/19 promoters

Gene	Gene-specific primer (5’ to 3’)	Nested gene-specific primer (5’ to 3’)
DkERF9m	CTCATATATAAACGTTCCTTCACCACATGTA	AAGGAACGTTTATATATGAGACGCGGCGGC
DkERF10m	AGCTTTCGTTATAGTTAATATTTGCGACCC	TATTAACTATAACGAAAGCTCAATTTATGT
	CTTTGATACCAACCATTCGACCACGAC	AATGGTTGGTATCAAAGTGAGTAGTAA
DkERF19m-1	GGGTACCGAACGTTATTTGAATTCC	TAACGTTCGGTACCCAATTCGCCCTATAG
	ATCACAGCAAAACCCTCCTCCACACGTC	AGGAGGGTTTTGCTGTGATGACGACGTC
	AGTGAAAGGAGAAATAACCTCCCCATGG	GTTATTTCTCCTTTCACTTCTTGTTCTT
DkERF19m-2	GAAAGCTTTATCCAGAGCTGAAAGAGAG	GCTCTGGATAAAGCTTTCGTCTTCATTCA

Table S8. A summary on AHCA responsive transcription factors from persimmon fruit

Gene name	GenBank No.	N-end MC domain	Family	Responsive to deastringency treatment (95% CO_2, 4% N_2, 1% O_2)	Putative functions in fruit	Reference
DkERF1	JN256071	No	Ethylene response factor	Up-regulation		Min *et al*. J Exp Bot, 2012
DkERF4	JN256074	No	Ethylene response factor	Up-regulation		Min *et al*. J Exp Bot, 2012
DkERF5	JN256075	No	Ethylene response factor	Up-regulation		Min *et al*. J Exp Bot, 2012
DkERF6	JN256076	No	Ethylene response factor	Up-regulation		Min *et al*. J Exp Bot, 2012
DkERF8	JN256078	No	Ethylene response factor	Up-regulation	Softening	Wang *et al.* Plant Biotechnol J, 2017
DkERF9	JX117848	No	Ethylene response factor	Up-regulation	Deastringency	Min *et al*. J Exp Bot, 2012
DkERF10	JX145122	Yes	Ethylene response factor	Up-regulation	Deastringency	Min *et al*. J Exp Bot, 2012
DkERF11	KJ170911	No	Ethylene response factor	Up-regulation		Min *et al*. PLoS ONE, 2014
DkERF12	KJ170912	No	Ethylene response factor	Up-regulation		Min *et al*. PLoS ONE, 2014
DkERF13	KJ170913	No	Ethylene response factor	Up-regulation		Min *et al*. PLoS ONE, 2014
DkERF14	KJ170914	No	Ethylene response factor	Up-regulation		Min *et al*. PLoS ONE, 2014
DkERF15	KJ170915	No	Ethylene response factor	Up-regulation		Min *et al*. PLoS ONE, 2014
DkERF16	KJ170916	No	Ethylene response factor	Up-regulation	Softening	Wang *et al.* Plant Biotechnol J, 2017
DkERF17	KJ170917	No	Ethylene response factor	Up-regulation		Min *et al*. PLoS ONE, 2014
DkERF18	KJ170918	No	Ethylene response factor	Up-regulation	Deastringency	Min *et al*. PLoS ONE, 2014
DkERF19	KJ170919	No	Ethylene response factor	Up-regulation	Deastringency<br>Softening	Min *et al*. PLoS ONE, 2014<br>Wang *et al.* Plant Biotechnol J, 2017
DkERF20	KJ170920	No	Ethylene response factor	Up-regulation		Min *et al*. PLoS ONE, 2014
DkERF21	KJ170921	No	Ethylene response factor	Up-regulation	Deastringency	Min *et al*. PLoS ONE, 2014
DkERF22	KJ170922	No	Ethylene response factor	Up-regulation	Deastringency	Min *et al.* PLoS ONE, 2014
DkMYB5	KP875528	No	MYB	Up-regulation		Fang *et al.* Postharvest Biol Technol, 2016
DkMYB6	KP875529	No	MYB	Up-regulation	Deastringency	Fang *et al*. Postharvest Biol Technol, 2016

DkMYB7	KP875530	No	MYB	Up-regulation		Fang *et al*. Postharvest Biol Technol, 2016
DkMYB8	KP875531	No	MYB	Up-regulation		Fang *et al*. Postharvest Biol Technol, 2016
DkbHLH1	KY849612	No	bHLH	Up-regulation		This publication
DkbHLH2	KY849613	No	bHLH	Up-regulation		This publication
DkMYB9	KY849603	No	MYB	Up-regulation		This publication
DkMYB10	KY849604	No	MYB	Up-regulation	Deastringency	This publication
DkMYB11	KY849605	No	MYB	Up-regulation		This publication
DkMYB12	KY849606	No	MYB	Up-regulation		This publication
DkMYB13	KY849607	No	MYB	Up-regulation		This publication
DkRH2-1	KY849614	No	Ring finger protein	Up-regulation		This publication
DkRH2-2	KY849615	No	Ring finger protein	Up-regulation		This publication
DkGT3-1	KY849616	No	MYB-like	Up-regulation		This publication
DkAN1-1	KY849617	No	Zinc finger	Up-regulation		This publication
DkIAA1	KY849618	No	Auxin-responsive protein	Up-regulation		This publication
DkHSF1	KY849619	No	Heat shock factor	Up-regulation		This publication
